# Study protocol: the sleeping sound with attention-deficit/hyperactivity disorder project

**DOI:** 10.1186/1471-2431-10-101

**Published:** 2010-12-30

**Authors:** Emma Sciberras, Daryl Efron, Bibi Gerner, Margot Davey, Fiona Mensah, Frank Oberklaid, Harriet Hiscock

**Affiliations:** 1 Centre for Community Child Health, Murdoch Childrens Research Institute, Royal Children's Hospital, Flemington Road, Parkville, Victoria, Australia; 2 Centre for Community Child Health, Murdoch Childrens Research Institute, Royal Children's Hospital, Victoria, Australia; Department of Paediatrics, University of Melbourne, Victoria, Australia; 3 Centre for Community Child Health, Murdoch Childrens Research Institute, Royal Children's Hospital, Flemington Road, Parkville, Victoria, Australia; 4 Melbourne Children's Sleep Unit, Monash Medical Centre, Clayton South, Victoria, Australia; 5 Clinical Epidemiology and Biostatistics Unit, Murdoch Childrens Research Institute, Royal Children's Hospital, Flemington Road, Parkville, Victoria, Australia; 6 Centre for Community Child Health, Murdoch Childrens Research Institute, Royal Children's Hospital, Victoria, Australia; Department of Paediatrics, University of Melbourne, Victoria, Australia; 7 Centre for Community Child Health, Murdoch Childrens Research Institute, Royal Children's Hospital, Victoria, Australia; Department of Paediatrics, University of Melbourne, Victoria, Australia

## Abstract

**Background:**

Up to 70% of children with Attention-Deficit/Hyperactivity Disorder (ADHD) experience sleep problems including difficulties initiating and maintaining sleep. Sleep problems in children with ADHD can result in poorer child functioning, impacting on school attendance, daily functioning and behaviour, as well as parental mental health and work attendance. The Sleeping Sound with ADHD trial aims to investigate the efficacy of a behavioural sleep program in treating sleep problems experienced by children with ADHD. We have demonstrated the feasibility and the acceptability of this treatment program in a pilot study.

**Methods/Design:**

This randomised controlled trial (RCT) is being conducted with 198 children (aged between 5 to 12 years) with ADHD and moderate to severe sleep problems. Children are recruited from public and private paediatric practices across the state of Victoria, Australia. Upon receiving informed written consent, families are randomised to receive either the behavioural sleep intervention or usual care. The intervention consists of two individual, face-to-face consultations and a follow-up phone call with a trained clinician (trainee consultant paediatrician or psychologist), focusing on the assessment and management of child sleep problems. The primary outcome is parent- and teacher-reported ADHD symptoms (ADHD Rating Scale IV). Secondary outcomes are child sleep (actigraphy and parent report), behaviour, daily functioning, school attendance and working memory, as well as parent mental health and work attendance. We are also assessing the impact of children's psychiatric comorbidity (measured using a structured diagnostic interview) on treatment outcome.

**Discussion:**

To our knowledge, this is the first RCT of a behavioural intervention aiming to treat sleep problems in children with ADHD. If effective, this program will provide a feasible non-pharmacological and acceptable intervention improving child sleep and ADHD symptoms in this patient group.

**Trial Registration:**

Current Controlled Trials ISRCTN68819261.

ISRCTN: ISRCTN68819261

## Background

Attention-Deficit/Hyperactivity Disorder (ADHD) is one of the most common developmental disorders in childhood, affecting approximately 5% of children and adolescents [1]. ADHD is characterised by pervasive levels of inattention, impulsivity and/or hyperactivity, resulting in impairment in school and/or home settings [2]. In addition to the symptomatic burden on the child and consequences for families and schools, ADHD is associated with huge financial costs. In the United States, the estimated annual societal cost of ADHD in childhood and adolescence is US$42.5 billion [3]. The mainstay of treatment for ADHD is stimulant medication. However, concerns have been expressed about the potential adverse effects of stimulant medication including poor growth and cardiac effects [4]. Any strategies which reduce the need for medication would therefore be regarded as desirable [4]; identifying and managing sleep problems might be one such strategy.

### Sleep problems in children with ADHD - prevalence, aetiology, and burden

Up to 70% of parents of children with ADHD report difficulties with their child's sleep [5], including difficulties initiating and maintaining sleep [6-9]. A recent meta-analysis found that children with ADHD have higher rates of parent-reported sleep problems than non-ADHD controls; problems include bedtime resistance, sleep onset difficulties, night awakenings and daytime sleepiness [10]. In addition, children with ADHD have sleep problems when using objective measures (e.g., polysomnography, actigraphy), including greater sleep onset latency and poorer sleep efficiency [10].

The cause of sleep problems in children with ADHD is multi-factorial. Medication may play a role, since insomnia is a well-known side-effect of stimulant medication [11] and bedtime refusal may be due to a rebound effect when stimulant medication wears off [12]. However, unmedicated children with ADHD also experience sleep problems [7,9,13]. Comorbid disorders may also contribute to sleep problems. Oppositional defiant disorder (ODD) and conduct disorder (CD) may lead to bedtime resistance [14-16], though at least one study has reported that ODD was unrelated to sleep problems in children with ADHD - this study reported that children with comorbid internalising difficulties such as anxiety were at a greater risk for sleep problems [17].

In 2006, we conducted a survey of 239 school-aged children with ADHD investigating the burden of sleep problems for these children [5]. Moderate/severe sleep problems were reported by 45% of parents and associated with significantly poorer child psychosocial quality of life using the Pediatric Quality of Life Inventory (coefficient -11.95, 95% CI (-15.82 to -8.08)), daily functioning using the Daily Parent Rating of Evening and Morning Behaviour (coefficient -7.72, 95% CI (5.78 to 9.66)), caregiver depression/anxiety/stress using the Depression Anxiety Stress Scale (odds ratio 3.15, 95% CI (1.65 to 6.02)), and family functioning (all p ≤ 0.01). All associations held after adjusting for confounders (medication use, comorbidities, child age, child gender, and socioeconomic status). Importantly, when compared to children without sleep problems, those with sleep problems were more likely to regularly miss (78 vs 54%) or be late for school (58 vs 34%), and their caregivers were more likely to be late to work (20% vs 4%).

### Treating sleep problems in children with ADHD

The majority of sleep problems in children with ADHD occur at or around sleep onset, and are behavioural in origin [5]. Behavioural sleep problems are amenable to intervention in the general population [18], and the same may be true in children with ADHD. However no controlled trials of behavioural sleep interventions have been reported in children with ADHD. Only one small study (n = 3) has evaluated behavioural interventions for sleep problems in unmedicated children with ADHD [19]. The intervention ran over five weeks using a written manual, with weekly telephone follow-up after the parent read each of the five chapters. Immediately following the intervention and at three months follow-up, the severity of the child's sleep problem decreased; the children's ADHD symptoms were unchanged.

Small, uncontrolled studies have evaluated the effects of medication on sleep problems in children with ADHD. The most common of these investigated the effects of clonidine, an alpha-agonist which causes drowsiness. In a chart review of 62 children and adolescents treated with clonidine, 85% showed some improvement in time to fall asleep, though one third of the sample experienced adverse effects including morning sedation [20]. There are significant concerns about accidental poisoning with clonidine [21]. Melatonin is a hormone secreted by the pineal gland soon after the onset of darkness. One open-labelled and two randomised controlled studies have examined the effectiveness of melatonin in the treatment of sleep-onset insomnia in ADHD. These found some effectiveness in reducing sleep onset latency [22,24], but the studies were small (n = 24-27) and none led to changes in the children's ADHD symptoms.

It could be argued that sleep problems in children with ADHD may be less amenable to change because of their other challenging behaviours, but trials in children with other challenging behaviours (ie learning disabilities and autism) demonstrated that behavioural sleep interventions can be effective [25-27]. If we could effectively manage sleep problems in children with ADHD then we may be able to improve child ADHD symptoms, quality of life and school attendance, as well as parent mental health and work attendance. However, in our survey only 45% of parents reported that their child's treating paediatrician had asked about the child's sleep and, of these, only 60% reported receiving advice. Forty percent of parents receiving advice reported that this was helpful [5].

In 2008, we developed a behavioural sleep intervention program for children with ADHD and moderate/severe sleep problems (n = 27). We evaluated the feasibility and helpfulness of a brief (1 session) versus extended (2 to 3 session) behavioural sleep program and explored the impact of each on child and family outcomes. At five months, both brief and extended groups reported a reduction in child sleep problems and ADHD symptoms (both 67%). There was a trend towards improved child psychosocial quality of life and daily functioning and parental anxiety in the extended group only (Cohen's *d*: 0.39, 0.47 and 0.50, respectively). Given the additional improvement in child and family outcomes for children in the extended group, we have selected this intervention to test in a fully-powered randomised controlled trial.

### Aims and hypotheses

We aim to conduct the first randomised controlled trial of a behavioural sleep intervention in primary school aged children (5-12 years) with ADHD, in order to determine whether managing sleep problems can effectively improve ADHD symptoms, child sleep, and other child and family outcomes. We hypothesise that, compared to the control group, benefits of a behavioural sleep intervention at 3, 6 and 12 months post-intervention will include:

1. Improved child outcomes:

a. Lower proportion with sleep problems (parent report).

b. Decreased sleep latency and increased sleep duration (actigraphy).

c. Lower (better) mean score on an ADHD symptom scale, as reported by parents and teachers independently (primary outcome).

d. Improved mean scores on continuous measures of working memory, behaviour (parent and teacher report), health-related quality of life, and school attendance.

2. Improved primary caregiver outcomes:

a. Lower proportion of mental health problems as measured on an adult mental health scale.

b. Improved mean scores on continuous measure of work attendance.

## Methods/Design

### Overall study design

**Figure 1:**
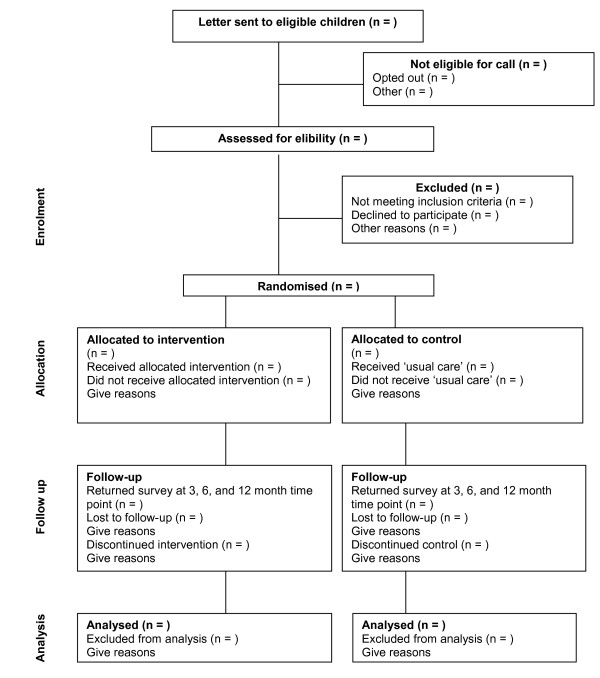
**Participant flow**.

The Sleeping Sound with ADHD project is an RCT of a behavioural sleep intervention versus usual care (see Figure 1). The project runs from mid 2010 to the end of 2012. This period encompasses participant recruitment (including determining eligibility for the study), baseline data collection, intervention delivery and participant follow-up (3-12 months). This project has been funded by the National Health and Medical Research Council of Australia (project grant number: 607362) and has been granted ethics approval by the Royal Children's Hospital (#30033) and the Victorian Department of Education and Early Childhood Development (#2010_000573) Human Research Ethics Committees.

### Participants

Participants include families of children aged 5 to 12 years with paediatrician diagnosed ADHD (any subtype) and at least one of the following sleep problems: sleep onset association disorder, limit setting disorder, delayed sleep phase or insomnia (idiopathic or psychophysiological), as defined by the American Academy of Sleep Medicine (see below). Children are also eligible if they have parent-reported night time anxiety (defined as having both difficulties falling asleep and significant worry at bedtime).

### Recruitment - Stage 1

Victorian paediatricians (public and private) pre-identify their patients with ADHD either through their medical software (eg Genie, Medical Director) or through case notes. Paediatricians send a study-designed letter to the child's primary caregiver inviting them to take part in the study. The letter advises parents that the research team will phone them to ask about their child's sleep and ADHD symptoms. An 'opt out' approach is used, whereby parents are asked to contact the study team if they *do not *wish to learn more about the study. If parents do not opt out within a two week period, the paediatrician provides the research team with the contact details of the families by fax or post. The team then contacts the parent to explain the study further. In our 2006 survey, this approach resulted in a 74% uptake rate and was well accepted by parents [5].

### Recruitment - Stage 2

The research team telephones all parents who do not opt out to assess inclusion/exclusion criteria (see section below) and whether or not the parent is interested in participating in the study (10 minute phone call). Eligible families are mailed an information sheet, consent form, and baseline survey. To determine the extent of any participant bias, data are collected and compared with non-participants; this includes child gender, child age, and socioeconomic status of the family's immediate neighbourhood.

### Inclusion criteria

Eligible families include children who meet all of the following criteria:

a) Full diagnostic criteria for ADHD, using the 18-item DSM ADHD Rating Scale IV, which is a validated scale measuring the core symptoms of ADHD [28]. We also use study designed questions to assess ADHD symptom duration ('Did your child have these symptoms for six months or longer before he/she was diagnosed with ADHD?), onset ('Did your child have these symptoms before he/she turned seven?) and impairment (Are these symptoms present at home, school or when out socially e.g., in the park, visiting friends)?

b) Moderate/severe sleep problems by parent report [5].

c) American Academy of Sleep Medicine diagnostic criteria for at least one sleep disorder including sleep onset association disorder, limit setting disorder, delayed sleep phase and/or insomnia (idiopathic or psychophysiological) [29]. Children experiencing significant night time anxiety and difficulty falling asleep at night are also eligible.

### Exclusion criteria

Children are excluded from the study if they meet any of the following criteria:

a) Receiving specialised help for their child's sleep from a psychologist or a specialised sleep clinic (apart from their paediatrician).

b) Have a serious medical condition (e.g., severe cerebral palsy) or an intellectual disability (IQ < 70). Children with other developmental or mental health comorbidities are not excluded.

c) Have suspected obstructive sleep apnoea (OSA). OSA is assessed using the three OSA items from the Child Sleep Habit's Questionnaire (CSHQ) [30]. This scale can help identify children who may suffer from OSA. Parents who report that their child sometimes or usually snores, stops breathing and/or snorts/gasps during their sleep are contacted by the lead investigator (HH), a paediatrician, for further assessment. If OSA is suspected, these children are excluded and referred to appropriate clinical services. After approximately six months, we will follow-up with children excluded due to suspected OSA to see whether they are eligible to participate in the study following clinical assessment and possible treatment i.e. if they have ongoing behavioural sleep problems despite treatment of their OSA.

### Sample size

The primary outcome is ADHD symptom scores. In order to detect a 0.4 standard deviation (clinically meaningful) shift in the mean ADHD score between the treatment and control group at the three-month follow-up, with 80% power and at a significance level of 0.05, we would require 99 children in each arm (198 total children). Allowing for up to 20% reduction in the proportion of children with sleep problems amongst the control arm, this sample size would also ensure adequate power to detect a further 20% reduction in the proportion of children with sleep problems between the intervention and control arms. It would also enable us to detect a shift in other secondary outcomes, such as the mean quality of life scores between the two treatment arms.

In order to have follow-up data on 198 children at the 3 month follow-up, we need paediatricians to send study information to approximately 1124 children with ADHD. This assumes an initial 70% response rate (i.e. consenting to hearing more about the study, n = 787), and that of these, 45% will have a moderate/severe sleep problems (based on 2006 survey [5], n = 354). This also takes into account 70% of those with moderate/severe sleep problems consenting to taking part in the study (n = 248) and 20% participant drop out over the follow-up period.

### Randomisation

Upon receiving the completed consent form and baseline survey, an independent research assistant randomises families to either the behavioural sleep intervention group or the control group of 'usual care'. Families are randomised using a pre-generated random number sequence developed by a statistician, which is contained in sealed opaque envelopes stored in the independent research assistant's office. We used varying block sizes of 2, 4 and 6 in the randomisation sequence to maintain balance between the trial arms over the course of the trial and so that allocations could not be predicted from the previous sequence. It is expected that the patient group will predominantly be boys, so that randomisation is stratified by gender in order for distribution proportions of males and females to be comparable across the randomisation groups. All families are mailed a letter to inform them of their group allocation. Intervention families are then telephoned to book a consultation time at their paediatrician's office. Control families can access usual care for ADHD from their child's paediatrician - our previous survey suggests this does not routinely involve sleep management [5]. If the parent consented to teacher participation, we mail the child's teacher a baseline survey to complete at the point of randomisation.

### Intervention group

The behavioural intervention is evidence based [18] and consists of two face-to-face, one-on-one sleep consultations with a trained clinician (trainee consultant paediatrician or psychologist) held two weeks apart and one follow-up telephone call another two weeks later. The first session focuses on an assessment of the child's sleep problem, providing information about normal sleep and sleep cycles, advice about sleep hygiene, and a plan specifically tailored to the child's particular sleep disorder. For example, sleep onset association disorder, typically associated with the need for parental presence at sleep time, is managed with adult fading (i.e., graduated extinction). This technique requires gradual withdrawal of parental presence from the child's bedroom over 7-10 days. Limit setting disorder is managed by ignoring child protests and rewarding compliance with bedtime routines. Delayed sleep phase is managed by temporarily setting the child's bedtime later, gradually bringing it forward, and waking the child at a pre-set time in the morning and encouraging early morning light exposure [18]. Parents are offered a range of management strategies and are free to choose the strategies they would like to try. All parents are also asked to complete a sleep diary. The second session is held two weeks later to review the sleep diary, reinforce strategies, trouble shoot and monitor progress.

A standardised consultation record is kept for all children, as per our pilot. This includes the presenting sleep problem/s, possible contributors to the problem (eg TV in bedroom), ADHD medication use, comorbidities, and usual bedtime routines. The clinician records the duration of each consultation, sleep problem diagnoses, family sleep management goals, handouts given to parents, and management strategies chosen by the family.

A follow-up telephone call is made at a time convenient for the family, approximately two weeks after the second consultation, to provide an opportunity for the child's parents to ask further questions, and to reinforce strategies, trouble shoot, and monitor progress.

Our pilot study demonstrated that this program is feasible to deliver and acceptable to families. However, a barrier to participation was travel to the Royal Children's Hospital Melbourne and we have addressed this by conducting consultations at the treating paediatrician's consulting rooms.

### Measures

Data are collected using parent, teacher and self-report questionnaires, as well as objective measures including a face-to-face assessment of working memory and actigraphy - all measures are outlined in Table 1. Outcomes are measured at 3, 6 and 12 months post randomisation with the exception of actigraphy (3 months only), the working memory test (6 months only) and teacher report (3 and 6 months only).

**Table 1:** Study measures and time-points

Measures		Time point			
		Baseline	3 months	6 months	12 months
ADHD IV Rating Scale	Parent	■	■	■	■
	Teacher	■	■	■	
Moderate/severe sleep problem	Parent	■	■	■	■
Children's Sleep Habits Questionnaire	Parent	■	■	■	■
Actiwatch 2	Child	■	■		
Strengths and Difficulties Questionnaire	Parent	■	■	■	■
	Teacher	■	■	■	
Pediatric Quality of Life Inventory	Parent	■	■	■	■
	Child			■	
School attendance	Parent	■	■	■	■
Working Memory Test Battery for Children	Child			■	
Other sleep help	Parent		■	■	■
Sleep program evaluation (intervention only)	Parent		■		
Depression Anxiety Stress Scale	Parent	■	■	■	■
Work attendance	Parent	■	■	■	
Anxiety Disorders Interview Schedule for Children IV	Parent	■			

#### Primary outcome

Child ADHD symptoms, using the ADHD Rating Scale IV- parent and teacher versions. An 18-item validated scale measuring the core symptoms of ADHD (inattention, impulsivity, hyperactivity), which has been demonstrated to be sensitive to change [28].

### Secondary outcomes

#### Child

Sleep problem - primary caregiver report of child sleep problem (none, mild, moderate or severe) [5,31].

Children's Sleep Habits Questionnaire (CSHQ) - 33-item, validated measure of disorders of initiating and maintaining sleep, which can distinguish clinical from community samples [30].

Actigraphy - The Actiwatch 2 (Philips Resprionics) is a small, motion sensor that is attached to the non dominant wrist to measure body movements and is used to provide an objective measure of sleep. Movement patterns are analysed and used to differentiate between sleep and wake times, thus providing an objective measure of sleep onset and total sleep duration. Use of actigraphy data in children has been shown to be reliable and valid and it also correlates well with data obtained using polysomnography (overnight observation of sleep) [32]. The Actiwatch is accompanied by an instruction sheet and sleep log which covers napping, medication use, time in bed, night awakenings, morning awakening time etc. The sleep log assists in the interpretation of Actiwatch data. Children wear the Actiwatch for seven days during school term to assess both weekday and weekend sleep behaviour.

Strengths and Difficulties Questionnaire (SDQ) - parent and teacher versions. A 25-item validated measure of behavioural and emotional problems for children aged 4 to 16 years. It provides standard scores on 5 subscales (hyperactivity/inattention, conduct problems, emotional symptoms, peer relationship problems, and prosocial behavior); a total problems score is derived from the first 4 subscales [33].

Pediatric Quality of Life Inventory (PedsQL - child and parent versions) - a 23-item validated measure for children aged 2 to 18 years. Provides total, physical, and psychosocial health summary scores, with higher scores indicating better health-related quality of life [34].

School attendance - number of days missed or late for school over the preceding three months [5].

Daily Parent Rating of Evening and Morning Behaviour (DREMB) scale - an 11-item rating of core ADHD symptoms and behavioural problems typically experienced over the past month [35].

Working Memory Test Battery for Children - a face-to-face assessment which provides an objective measure of the impact of the sleep intervention on the child's working memory, a critical executive function. Backwards Digit Recall, Counting Recall, and Listening Recall subtests are administered [36].

Other sleep help - parent report of other professional help sought for their child's sleep eg GP, school nurse.

Sleep program evaluation (intervention group only) - parent report of the usefulness of program strategies and ability to put strategies into practice.

#### Primary caregiver

Depression Anxiety Stress Scale (DASS) - a validated 21-item measure of adult mental health with clinical cut points for each of the three subscales of depression, anxiety and stress [37].

Work attendance - number of days missed or late for work over the preceding three months [5].

Socio-demographic questions are also included in the baseline questionnaire - these cover family composition, parental education and age, language spoken at home, annual household income, and child medication use and diagnosed comorbidities.

A comprehensive assessment of comorbid diagnoses is completed using the Anxiety Disorders Interview Schedule for Children (ADIS-C-IV).^38 ^This assessment is conducted over the telephone shortly after randomisation (the ADIS-C-IV is validated for administration over the telephone) [38]; assessing comorbidity is important in understanding any differential effects of the intervention as a result of the child's comorbidity. We will also assess inter-rater reliability to ensure consistency in the way that interviewers code parental responses on the ADIS-C. We will audio record the first 10 interviews of parents who give verbal consent; interviewers will code parental responses from these ten interviews and inter-rater reliability coefficients will be calculated.

### Data analysis

Analyses will be by 'intention to treat' at the level of the individual child. At the 3, 6 and 12 month follow-up, we will compare change in mean primary caregiver and teacher scores (3 and 6 month follow-up only) on the ADHD Rating Scale IV between the two trial arms using t tests. We will also compare proportions of children with moderate/severe vs no/mild sleep problems between the two arms using chi squared analysis at each of the follow-up periods. We will compare mean scores at follow up for sleep latency and duration (actigraphy), child working memory, behaviour, health-related quality of life, daily functioning and school attendance, as well as compare mean number of days late for work and parent mental health (depression, anxiety and stress) between the two arms, using either Mann-Whitney or t tests, as dictated by data distribution.

At each follow-up, we will conduct a set of regression analyses (linear regression for continuous data, logistic regression for categorical data) for each outcome, adjusted for potential confounders identified a priori. Confounders will include child age, medication use, comorbidities, family socio-demographic factors, and family socioeconomic status, which will be assigned according to postal code of residence using the Index of Relative Socioeconomic Disadvantage (mean 1000, s.d. 100) from the Australian Bureau of Statistics census-based Socio-Economic Indexes for Areas (SEIFA) [39].

## Discussion

ADHD is common and frequently associated with sleep problems which are associated with poorer child and family outcomes [5]. Despite the high prevalence of behavioural sleep problems in children with ADHD, little research has focused on their clinical management. This study is the first RCT to investigate the efficacy of a behavioural sleep intervention in children with ADHD and moderate to severe sleep problems. We are investigating whether or not treating sleep problems in this population can improve ADHD symptoms and other child and family outcomes such as child school attendance and parent mental health. If this intervention is effective, we will be able to take the next steps in the program's evaluation and translation i.e. an effectiveness trial in an existing workforce. We have a mechanism to conduct such a trial in the secondary care setting through our 380-member Australian Paediatric Research Network (APRN) [40]. Through the APRN, child mental health services and the Royal Australasian College of Physicians, we can effect the wider translation of this program and its potential benefits to children with ADHD and their families.

## Abbreviations

ADHD: Attention-Deficit/Hyperactivity Disorder; ODD: Oppositional defiant disorder; CD: Conduct disorder; OSA: Obstructive sleep apnoea; CSHQ: Child Sleep Habit's Questionnaire; SDQ: Strengths and Difficulties Questionnaire; PedsQL: Pediatric Quality of Life Inventory; DREMB: Daily Parent Rating of Evening and Morning Behaviour; DASS: Depression Anxiety Stress Scale; ADIS-C-IV: Anxiety Disorders Interview Schedule for Children; APRN: Australian Paediatric Research Network; RCT: Randomised controlled trial.

## Competing interests

The authors declare that they have no competing interests.

## Authors' contributions

ES, HH, DE, FO contributed to the overall design and conception of the study and assisted with the writing of the grant application. ES also drafted this manuscript. BG contributed to implementing the trial and overseeing all stages. BG also reviewed and edited this manuscript. MD provided advice regarding measurement, assessment and treatment of sleep and assisted with the manuscript revision. FM contributed to the statistical design of the study and commented on this manuscript. All authors read and approved the final manuscript.

## Pre-publication history

The pre-publication history for this paper can be accessed here:

http://www.biomedcentral.com/1471-2431/10/101/prepub

## References

[B1] Polanczyk G de Lima MS Horta BL Biederman J Rohde LA The worldwide prevalence of ADHD: A systematic review and metaregression analysis Am J Psychiatry 2007 164 942 8 10.1176/appi.ajp.164.6.942 17541055

[B2] American Psychiatric Association Diagnostic and Statistical Manual of Mental Disorders 1994 4 Washington, DC: American Psychiatric Association

[B3] Pelham WE Foster M Robb JA The Economic Impact of Attention-Deficit/Hyperactivity Disorder in Children and Adolescents J Pediatr Psychol 2007 32 711 27 10.1093/jpepsy/jsm022 17556402

[B4] Benner-Davis S Heaton PC Attention deficit and hyperactivity disorder: controversies of diagnosis and safety of pharmacological and nonpharmacological treatment Curr Drug Saf 2007 2 33 42 10.2174/157488607779315444 18690948

[B5] Sung V Hiscock H Sciberras E Efron D Sleep problems in children with attention-deficit/hyperactivity disorder - Prevalence and the effect on the child and family Arch Pediatr Adolesc Med 2008 162 336 42 10.1001/archpedi.162.4.336 18391142

[B6] Greenhill L Puigantich J Goetz R Hanlon C Davies M Sleep Architecture and rem-sleep measure in prepubertal children with attention deficit disorder with hyperactivity Sleep 1983 6 91 101 6878986 10.1093/sleep/6.2.91

[B7] Kaplan BJ McNicol J Conte RA Moghadam HK Sleep disturbance in preschool-aged hyperactive and nonhyperactive children Pediatrics 1987 80 839 44 3684394

[B8] Ball JD Tiernan M Janusz J Furr A Sleep patterns among children with attention-deficit hyperactivity disorder: A reexamination of parent perceptions J Pediatr Psychol 1997 22 389 98 10.1093/jpepsy/22.3.389 9212555

[B9] Owens JA Maxim R Nobile C McGuinn M Msall M Parental and self-report of sleep in children with attention-deficit/hyperactivity disorder Arch Pediatr Adolesc Med 2000 154 549 55 10850500 10.1001/archpedi.154.6.549

[B10] Cortese S Faraone SV Konofal E Lecendreux M Sleep in Children With Attention-Deficit/Hyperactivity Disorder: Meta-Analysis of Subjective and Objective Studies J Am Acad Child Adolesc Psychiatry 2009 48 894 908 10.1097/CHI.0b013e3181ae09c9 19625983

[B11] Barkley RA A review of stimulant drug research with hyperactive children J Child Psychol Psychiatry 1977 18 137 65 10.1111/j.1469-7610.1977.tb00425.x 326801

[B12] Barkley RA McMurray MB Edelbrock CS Robbins K Side effects of methylphenidate in children with Attention Deficit Hyperactivity Disorder: A systematic, placebo-controlled evaluation Pediatrics 1990 86 184 92 2196520

[B13] Owen J Sangal JM Sutton V Bakken R Allen A Kelsey D Objective and subjective measures of sleep in children with attention-deficit/hyperactivity disorder Sleep Med 2009 10 446 56 10.1016/j.sleep.2008.03.013 18693137

[B14] Biederman J Newcorn L Sprich S Comorbidity of attention deficit hyperactivity disorder with conduct, depressive, anxiety, and other disorders Am J Psychiatry 1991 148 564 77 2018156 10.1176/ajp.148.5.564

[B15] Frick PJ Kamphaus RW Lahey B Loeber R Christ MAG Hart EL Academic underachievement and the disruptive behaviour disorders Journal of Consulting and Clinical Psychology 1991 59 289 94 10.1037/0022-006X.59.2.289 2030190

[B16] Barkley RA Attention-Deficit Hyperactivity Disorder: A Handbook for Diagnosis and Treatment 2006 New York: The Guilford Press

[B17] Mayes SD Calhoun SL Bixler EO Fauzia Mahr VAN Hillwig-Garcia J Elamir B ADHD Subtypes and Comorbid Anxiety, Depression, and Oppositional-Defiant Disorder: Differences in Sleep Problems J Pediatr Psychol 2009 34 328 37 10.1093/jpepsy/jsn083 18676503 PMC2722128

[B18] Mindell JA Owen J A Clinical Guide to Pediatric Sleep Diagnosis and Management of Sleep Problems in Children and Adolescents 2003 Philadelphia, PA: Lippincott Williams & Wilkins

[B19] Mullane J Corkum P Case Series: Evaluation of a behavioural sleep intervention for three children with attention-deficit/hyperactivity disorder and dyssomnia J Atten Disord 2006 10 217 27 10.1177/1087054706288107 17085633

[B20] Prince JB Wilens TE Biederman J Spencer TJ Wozniak JR Clonidine for sleep disturbances associated with attention-deficit hyperactivity disorder: A systematic chart review of 62 cases J Am Acad Child Adolesc Psychiatry 1996 35 599 605 10.1097/00004583-199605000-00014 8935206

[B21] Sinha Y Cranswick NE Clonidine poisoning in children: A recent experience J Paediatr Child Health 2004 40 678 80 10.1111/j.1440-1754.2004.00491.x 15569283

[B22] Weiss MD Wasdell MB Bomben MM Rea KJ Freeman RD Sleep hygiene and melatonin treatment for children and adolescents with ADHD and initial insomnia J Am Acad Child Adolesc Psychiatry 2006 45 512 9 10.1097/01 chi.0000205706.78818.ef 16670647

[B23] Tjon Pian G CV Broeren JPA Starreveld JS Versteegh FGA Melatonin for treatment of sleeping disorders in children with attention deficit/hyperactivity disorder: a preliminary open label study Eur J Pediatr 2003 162 554 5 10.1007/s00431-003-1207-x 12783318

[B24] Van Der Heijden KB Smits MG Van Someren EJW Ridderinkhof R Boudewijn Gunning W Effect of melatonin on sleep, behavaiour, and cognition in ADHD and chronic sleep-onset insmonia J Am Acad Child Adolesc Psychiatry 2007 46 233 41 10.1097/01.chi.0000246055.76167.0d 17242627

[B25] Kodak T Piazza CC Assessment and behavioural treatment of feeding and sleeping disorders in children Autism Spectrum Disorders Child Adolesc Psychiatr Clin N Am 2008 17 887 905 10.1016/j.chc.2008.06.005 18775376

[B26] Montgomery P Stores G Wiggs L The relative efficacy of two brief treatments for sleep problems in young learning disabled (mentally retarded) children: a randomised controlled trial Arch Dis Child 2004 89 125 30 10.1136/adc.2002.017202 14736626 PMC1719807

[B27] Cortesi F Giannotti F Ivanenko A Johnson K Sleep in children with autistic spectrum disorder Sleep Med 2010 11 659 64 10.1016/j.sleep.2010.01.010 20605110

[B28] DuPaul GJ Power TJ Anastopoulos AD Reid R ADHD Rating Scale IV: Checklists, Norms, and Clinical Interpretation 1998 New York, NY: The Guilford Publications Inc

[B29] American Academy of Sleep Medicine The International Classification of Sleep Disorders, Revised: Diagnostic and Coding Manual 2001 Westchester, USA: American Academy of Sleep Medicine

[B30] Owens J Spirito A McGuinn M The Children's Sleep Habits Questionnaire (CSHQ): Psychometric properties of a survey instrument for school aged children Sleep 2000 23 1043 51 11145319

[B31] Hiscock H Canterford L Ukoummunne OC Wake M Adverse associations of sleep problems in Australian preschoolers: National Population Study Pediatrics 2007 119 86 93 10.1542/peds.2006-1757 17200274

[B32] Sadeh A Hauri P Kripke D Lavie P The role of actigraphy in the evaluation of sleep disorders Sleep 1995 18 288 302 7618029 10.1093/sleep/18.4.288

[B33] Goodman R Psychometric Properties of the Strengths and Difficulties Questionnaire J Am Acad Child Adolesc Psychiatry 2001 40 1337 45 10.1097/00004583-200111000-00015 11699809

[B34] Varni JW Limbers CA Burwinkle TM Parent proxy-report of their children's health-related quality of life: an analysis of 13,878 parents' reliability and validity across age subgroups using the PedsQL™ 4.0 Generic Core Scales Health and Quality of Life Outcomes 2007 5 1043 51 10.1186/1477-7525-5-2 PMC1769359 17201923

[B35] Kelsey DK Sumner CR Casat CD Once-daily atomoxetine treatment for children with attention-deficit/hyperactivity disorder, including an assessment of evening and morning behaviour: a double-blind placebo-controlled trial Pediatrics 2004 114 e1 e8 10.1542/peds.114.1.e1 15231966

[B36] Gathercole S Pickering S Assessment of Working Memory in Six- and Seven-Year-Old Children J Educ Psychol 2000 92 377 90 10.1037/0022-0663.92.2.377

[B37] Lovibond PH Lovibond SH The structure of negative emotional states: comparison of the Depression Anxiety Stress Scales (DASS) with the Beck Depression and Anxiety Inventories Behaviour Research and Therapy 1995 33 335 43 10.1016/0005-7967(94)00075-U 7726811

[B38] Lyneham HJ Rapee RM Agreement Between Telephone and In-Person Delivery of a Structured Interview for Anxiety Disorders in Children J Am Acad Child Adolesc Psychiatry 2005 44 274 82 10.1097/00004583-200503000-00012 15725972

[B39] Australian Bureau of Statistics Census of Population and Housing: Socio-Economic Indexes for Areas (SEIFA), Australia - Data only 2008 Canberra: Australian Bureau of Statistics http://www.abs.gov.au/AUSSTATS/abs@.nsf/DetailsPage/2033.0.55.0012006?OpenDocument

[B40] Hiscock H Efron D Wasserman R Wake M Power to the paediatricians The Australian Paediatric Research Network is born J Paediatr Child Health 2010 20598074 10.1111/j.1440-1754.2010.01772.x

